# Allelic Association, DNA Resequencing and Copy Number Variation at the Metabotropic Glutamate Receptor *GRM7* Gene Locus in Bipolar Disorder

**DOI:** 10.1002/ajmg.b.32239

**Published:** 2014-05-08

**Authors:** Radhika Kandaswamy, Andrew McQuillin, David Curtis, Hugh Gurling

**Affiliations:** 1 Molecular Psychiatry Laboratory, Mental Health Sciences Unit, Faculty of Brain Sciences, University College LondonLondon, UK; 2 Department of Psychological Medicine, St. Bartholomew's and the Royal London School of Medicine and Dentistry, Queen Mary University of LondonLondon, UK

**Keywords:** genetic, case–control, allelic association study, 3′-UTR, CNV

## Abstract

Genetic markers at the *GRM7* gene have shown allelic association with bipolar disorder (BP) in several case–control samples including our own sample. In this report, we present results of resequencing the *GRM7* gene in 32 bipolar samples and 32 random controls selected from 553 bipolar cases and 547 control samples (UCL1). Novel and potential etiological base pair changes discovered by resequencing were genotyped in the entire UCL case–control sample. We also report on the association between *GRM7* and BP in a second sample of 593 patients and 642 controls (UCL2). The three most significantly associated SNPs in the original UCL1 BP GWAS sample were genotyped in the UCL2 sample, of which none were associated. After combining the genotype data for the two samples only two (rs1508724 and rs6769814) of the original three SNP markers remained significantly associated with BP. DNA sequencing revealed mutations in three cases which were absent in control subjects. A 3′-UTR SNP rs56173829 was found to be significantly associated with BP in the whole UCL sample (*P* = 0.035; OR = 0.482), the rare allele being less common in cases compared to controls. Bioinformatic analyses predicted a change in the centroid secondary structure of RNA and alterations in the miRNA binding sites for the mutated base of rs56173829. We also validated two deletions and a duplication within *GRM7* using quantitative-PCR which provides further support for the pre-existing evidence that copy number variants at *GRM7* may have a role in the etiology of BP. © 2014 The Authors. American Journal of Medical Genetics Part B: Neuropsychiatric Published by Wiley Periodicals, Inc.

## INTRODUCTION

Bipolar disorder (BP) is diagnosed using operational criteria which define episodes of mania or hypomania with or without depression and are considered as reliable and valid as most medical diagnoses [Spitzer et al., 2012]. Bipolar and genetically related spectrum disorders such as unipolar affective disorders have a heritability of 89% with environmental effects limited to the specific or unique environment with no evidence for effects from the shared family environment [McGuffin et al., 2003].

Metabotropic glutamate receptors (mGluRs) are G protein coupled receptors which have a critical role in brain function. mGluRs are present in all regions of the brain involved in learning, memory, anxiety, and the perception of pain. They are highly expressed in pre- and postsynaptic neurons in the cerebellum, cerebral cortex, hippocampus, cingulate cortex, frontal cortex, amygdala, hippocampus, and locus coeruleus [Shigemoto et al., 1997; O'Connor et al., 2010]. mGluRs are involved in long-term potentiation and long-term depression and have a vital role in modulating glutamate release [Sanderson et al., 2011]. GRM7 is the most highly conserved group III metabotropic glutamate receptor which prevents risk of excitotoxicity in neurons presynaptically through inhibition of the second messenger adenylate cyclase and postsynaptically decreases NMDA receptor activity [Gu et al., 2012]. Presynaptic mGluR7 modulates the release of both l-glutamate and GABA and is involved in excitability levels in specific neuronal circuits, thereby influencing different emotional states such as anxiety and depression as well as cognitive dysfunction [Swanson et al., 2005].

*GRM7* is located on 3p26.1 and previous linkage studies have implicated this chromosomal region 3p in BP [Kelsoe et al., 2001; Fallin et al., 2005; Etain et al., 2006]. A recent comparative linkage meta-analysis of all whole genome linkage studies of BP found evidence of linkage at 3p25.3–3p22.1 using narrow (*P* = 0.0099) and broad spectrum (*P* = 0.0060) diagnostic models of BP [Tang et al., 2011]. Independent studies by Breen et al. [2011] and Pergadia et al. [2011] reported evidence for genetic linkage between major depressive disorder and chromosome 3p26–p25 with LOD scores of 4.0 and 4.14, respectively.

Genetic association between markers at the *GRM7* locus and BP was reported by the Wellcome Trust Case Control Consortium with the most significantly associated SNP at *P* = 0.000097. A significant association within any one genome wide association study (GWAS) has not been reported for *GRM7*. However, the involvement of *GRM7* as a bipolar affective disorder susceptibility locus has accumulated in several independent studies [WTCCC, 2007; Sklar et al., 2008; Alliey-Rodriguez et al., 2011]. A recent GWAS of personality traits in bipolar patients found association for a *GRM7* SNP with the neuroticism-anxiety scale of the Zuckerman–Kuhlman Personality Questionnaire (rs13080594, *P* = 7.68 × 10^−7^) [Alliey-Rodriguez et al., 2011]. A GWAS of major depression and meta-analyses have also demonstrated association with *GRM7* [Sullivan et al., 2009; Muglia et al., 2010; Shi et al., 2011; Shyn et al., 2011]. Comorbidity between bipolar I disorder and ADHD has been well documented previously [Faraone et al., 2012]. A Korean study reported association between the *GRM7* polymorphism rs37952452 and attention-deficit hyperactivity disorder (ADHD) using the transmission disequilibrium method in trios [Park et al., 2013]. The contribution of CNVs in the psychiatric disorders has been appreciated only recently with the implementation of array technologies that enable genome-wide determination of CNVs. Two studies of CNVs in BP reported no overall increase in CNV load in cases and found that controls had a significant excess of CNVs [Grozeva et al., 2010; McQuillin et al., 2011]. In a third study, a nominally significant increase in singleton CNVs in BP cases compared with controls was found [Zhang et al., 2009]. Deletions and duplications in *GRM7* were found in the UCL1 bipolar sample as well [McQuillin et al., 2011]. Rare CNVs occurring within *GRM7* have been documented in patients with other psychiatric disorder patients, such as mood disorder [Saus et al., 2010], schizophrenia [Walsh et al., 2008] and ADHD [Elia et al., 2012]. A growing body of evidence suggests a strong relationship between mGluR7 function and the action of antidepressants and mood-stabilizers [Palucha, 2006]. Fabbri et al. [2013] investigated the modulation of early antidepressant efficacy by glutamatergic gene variants in the STAR*D sample and found that a *GRM7* SNP rs1083801 was associated with early response under a recessive model. Evidence from genetic, pharmacological, and animal studies suggests a connection between *GRM7* and BP and other neuropsychiatric disorders. The main aim of this study was to build upon the positive allelic association studies of *GRM7* we previously published [Sklar et al., 2008] by resequencing exons and flanking regions of the *GRM7* gene in bipolar cases selected for inheriting a disease haplotype discovered in original GWAS dataset in order to find potential etiological base pair changes within *GRM7*. Any novel SNPs or mutations likely to be pathogenic were then genotyped in the UCL1 sample and a new independent bipolar sample (UCL2) in order to test for allelic association with BP. Coupled with this we also attempted to validate the three CNVs identified in the original UCL1 sample using QRT-PCR.

## MATERIALS AND METHODS

### UCL Bipolar Case–Control Sample

The study included 1,099 affected bipolar research subjects and 1,152 normal comparison subjects that were sampled in 2 cohorts. The first cohort called UCL1 comprised of 506 bipolar I cases and 510 normal comparison subjects which were subjected to a GWAS resulting in publications focusing on the top association hits [Sklar et al., 2008]. The second cohort (UCL2) comprised of 409 bipolar I (69%) and 184 bipolar II research subjects, and 642 normal control subjects. The sample of 1,152 normal comparison subjects comprised 672 who were screened for an absence of psychiatric disorders plus 480 unscreened British normal volunteers provided by European Collection of Animal Cell Cultures (ECACC). All cases and controls were selected to be of UK or Irish ancestry. UK National Health Service multicenter and local research ethics approvals were obtained and signed informed consent was given by all subjects. All UCL bipolar subjects and the psychiatrically screened control subjects were interviewed by a psychiatrist using the lifetime version of the Schizophrenia and Affective Disorders Schedule (SADS-L) [Spitzer et al., 1975]. The control subjects were selected if they were found to be normal after screening with the SADS-L interview and it was established that they had no first degree relatives with any mental disorder. All bipolar subjects were found to have BP diagnoses according to Research Diagnostic Criteria [Spitzer et al., 1975]. All the bipolar research subjects were also rated with the 90-item OPCRIT checklist [McGuffin et al., 1991]. DNA was obtained from blood samples for the cases and controls in UCL1 and from saliva samples for the cases in UCL2. DNA was extracted for all UCL samples using methods we have published previously [Pereira et al., 2011; Kandaswamy et al., 2013].

### Detection and Evaluation of New Variants

*GRM7* failed to reach genome-wide significance in the UCL1 BP GWAS and thus was not reported in the main findings of the study [Ferreira et al., 2008; Sklar et al., 2008]. A list of all the variants in *GRM7* genotyped in the UCL1 BP GWAS is reported in Supplementary Table SI and the most significantly associated *GRM7* marker was the SNP rs1508724 [*P* = 0.0028; OR (95% CI) = 1.33 (1.10–1.60)]. In the absence of any strongly associated haplotype, DNA samples from 32 bipolar research subjects homozygous or heterozygous for the rare allele of rs1508724 and also with an early age of onset of BP were selected for sequencing. Also, 32 random control subjects were selected for the sequencing of *GRM7*.

Sequencing was carried out on 15 exons included in all the *GRM7* isoforms (UCSC, March 2006 assembly), exon/intron boundaries, ∼3 kb of the promoter region of the isoforms ENST00000486284 (main isoform) and ENST00000463676, and 1 kb promoter region of the isoform ENST00000458641, 5′-UTR and 3′-UTR of the *GRM7* gene. The sequence of primers used for sequencing is listed in Supplementary Table SII. Sequencing was done using the Big Dye terminator v3.1 Cycle Sequencing kit (Applied Biosystems, Warrington, UK) on an ABI 3730*xl* DNA Analyzer (Applied Biosystems). Sequencing data was analyzed using the Staden Package [Staden, 1996]. Bioinformatic analysis to determine potentially functional SNPs was carried out using the UCSC genome browser (http://genome.ucsc.edu/), PolyPhen2 [Adzhubei et al., 2013] RESCUE-ESE (http://genes.mit.edu/burgelab/rescue-ese/) [Fairbrother et al., 2004], TESS (http://www.cbil.upenn.edu/cgi-bin/tess/tess), UTRsite (http://utrsite.ba.itb.cnr.it/) [Grillo et al., 2010], and RNAwebserver (rna.tbi.univie.ac.at/).

### Genotyping and Association Analysis

SNP genotyping for the three most significantly associated *GRM7* SNPs, rs1508724, rs11710946, and rs6769814 in the UCL1 BP GWAS was performed in the UCL2 samples by allele-specific PCR at KBiosciences (KBiosciences, Hoddesdon, UK). Rare variants or potentially etiological SNPs found by sequencing were genotyped in-house using KASPar (KBiosciences) and High resolution melting curve genotyping methods on a LightCycler 480 (Roche, West Sussex, UK) in both the UCL1 and UCL2 samples. The primers used are listed in Supplementary Table SIII. For all SNPs genotyped, 17% of samples were reduplicated to detect error and confirm the reproducibility of genotypes. All these data were analyzed to confirm Hardy–Weinberg equilibrium (HWE). Genotypic and allelic associations for SNPs were tested using *χ*^2^ tests. Haplotype tests of association were performed using Haploview [Barrett, 2009]. Exploratory association analysis with imputed SNP data was carried out on the combined UCL 1 and 2 data using PLINK [Purcell et al., 2007]. Data from the European samples in the 1000 genome project (20110521 genotype and haplotype release) was used as a reference panel. Significance values shown for all analyses are uncorrected for multiple testing and a cut-off significance value of *P* < 0.05 was used. We performed a gene-based burden test (C-alpha) [Neale et al., 2011] using PLINK/SEQ (http://atgu.mgh.harvard.edu/plinkseq/) that takes both protective and risk alleles into account for testing association with the disease. C-alpha test statistic *P*-value < 0.05 after 10,000 permutations was considered significant.

### CNV Validation Using QRT-PCR

CNVs detected in the UCL1 samples were validated using TaqMan® RNase P Copy Number Reference (CNR) Assay, Human (Applied Biosystems). In the duplex, real-time PCR experiment, a UPL primer-probe (Universal Probe Library, Roche Diagnostics Ltd., UK) combination for the detection of the target gene, *GRM7* (Supplementary Table SIV) and the TaqMan® RNase P CNR assay for the detection of a reference gene, RNase P was used. For the QRT-PCR reaction in a 384-well plate, 10 ng of template DNA, 0.2 µl each of 10 µM forward and reverse primers and 10 µM probe, 0.5 µl of RNase P CNR assay mix, 5 µl of LightCycler® probes master reaction mix and PCR water to a total volume of 10 µl was added. The cycling conditions used were 95°C for 10 min, and 40 cycles of 95°C for 15 sec and 60°C for 1 min. Data analysis was performed using LightCycler relative quantification method that is based on the ΔΔC_t_ method of QRT-PCR data analysis [Livak and Schmittgen, 2001].

## RESULTS

### Association Analysis

In our collaborative GWAS [Sklar et al., 2008], eight SNPs in *GRM7* showed allelic association with BP in the UCL1 sample and the most significantly associated SNP at this locus was rs1508724 (*P* = 0.0014) (Supplementary Table SI). We failed to replicate the association of the three most significantly associated UCL1 GWAS SNPs, rs1508724, rs11710946, and rs6769814 in the UCL2 sample (Table 1). On combining the UCL1 and UCL2 genotype data for the three SNPs using PLINK, two SNPs, rs1508724 and rs6769814, remained significant (Table 2). We also performed a meta-analysis to assess the association of these SNPs in the combined sample keeping account of heterogeneity. Only one SNP rs6769814 remained significant as a result of meta-analysis (Cochrane Q-statistic *P*-value of 0.2166, Fixed effects model; *P*-value = 0.03506 and OR = 1.1544) (Table 2). The heterogeneity index for the other two SNPs rs1508724 and rs11710946 was significantly high, 76.83 and 81.67, respectively. This suggested that the UCL1 and UCL2 are not completely homogeneous although both the samples were selected based on strict ancestry based questionnaire.

**TABLE I:** SNP Association Results With *GRM7* in the UCL Bipolar Samples

SNP	Position (NCBI35/hg19)	*UCL1*				*UCL2*			
	
		Allele counts (MAF)		*P*-value	OR	Alleles counts (MAF)		*P*-value	OR
	
		BP	CON			BP	CON		
rs1508724	7241745	A 344 (0.34)	A 280 (0.28)	**0.001**	1.36	A 297 (0.29)	A 270 (0.29)	0.979	1.00
		G 668 (0.66)	G 740 (0.72)			G 723 (0.71)	G 652 (0.71)		
rs11710946	7246241	A 359 (0.36)	A 430 (0.42)	**0.002**	0.75	A 399 (0.40)	A 351 (0.38)	0.634	1.05
		G 653 (0.64)	G 590 (0.58)			G 615 (0.60)	G 567 (0.62)		
rs6769814	7251433	G 349 (0.35)	G 297 (0.29)	**0.008**	1.29	G 324 (0.31)	G 275 (0.30)	0.571	1.06
		A 657 (0.65)	A 721 (0.71)			A 716 (0.69)	A 639 (0.70)		

MAF, minor allele frequency; BP, bipolar; CON, control; OR, odds-ratio; *P* < 0.05 in bold.

**TABLE II:** Combined Analysis of *GRM7* SNPs in UCL1 and UCL2 Samples

SNP	Position (NCBI35/hg19)	Combined analysis				Meta-analysis				Q	I^2^
	
		Allele counts (MAF)				Fixed effects model		Random effects model			
		
		BP	CON	*P*-value	OR	*P*-value	OR	*P*-value	OR		
rs1508724	7241745	A 623 (0.31)	A 550 (0.28)	**0.043**	1.15	0.032	1.16	0.32	1.15	0.038	76.83
		G 1373 (0.69)	G 1392 (0.72)								
rs11710946	7246241	A 751 (0.38)	A 781 (0.40)	0.092	0.90	0.080	0.89	0.477	0.90	0.020	81.67
		G 1241 (0.62)	G 1157 (0.60)								
rs6769814	7251433	G 652 (0.33)	G 572 (0.30)	**0.045**	1.15	0.035	1.15	0.091	1.15	0.217	34.5
		A 1354 (0.67)	A 1360 (0.70)								

MAF, minor allele frequency; BP, bipolar; CON, control; OR, odds ratio; Q, *P*-value for Cochrane's Q statistic; I^2^, I^2^ heterogeneity index (0–100); *P* < 0.05 in bold.

*P*-values in fixed effects model column <0.05 need to be in bold.

### Sequencing

Detection of novel variants in *GRM7* associated with BP was pursued by resequencing all the exons in all the *GRM7* isoforms, exon/intron boundaries, ∼3 kb of the promoter region of the isoforms ENST00000486284 and ENST00000463676, and 1 kb of the promoter region of the isoform ENST00000458641, 5′-UTR and 3′-UTR of the *GRM7* gene in 32 bipolar cases and 32 random controls. In total, we found 71 previously published and 10 novel mutations in *GRM7* (Supplementary Table SV). Most of the published SNPs were of similar frequency in the sequenced cases and controls (Supplementary Table SV), therefore, were not genotyped further. Also, SNPs that were in complete LD with the UCL1 BP GWAS SNPs were not genotyped in the complete sample. Of the novel mutations, three were single base substitutions, two STRs, and five indels (Supplementary Table SV). Bioinformatic analysis was carried out for the novel SNPs to predict their effect on the structure and/or function of *GRM7*. Finally, based on increased frequency in cases compared to controls in the sequencing panel, 18 SNPs were further genotyped in the complete UCL sample (UCL1 and UCL2). Among these, only rs56173829 was significantly associated with BP in our sample (*P* = 0.035; OR = 0.4829) (Table 3), although it was less common in cases than controls and did not survive multiple testing. The rare allele of the SNP rs56173829 was present in 25/1821 alleles (1.4%) in control subjects versus 12/1810 (0.66%) in bipolar cases (Table 3). The SNP rs56173829 is present in the 3′-UTR of the long isoform of *GRM7* and may play an important role in microRNA (miRNA) binding.

**TABLE III:** Tests of Allelic Association of GRM7 SNPs Found by Resequencing, in the UCL Sample

SNP	Position (NCBI35/hg19)	Min/Maj allele	MAF	Genotype counts	Allele counts	*P*-value	OR (95% CI)
rs114774914	6901783						
Cases		A/T	0.014	0/26/897	26/1820	0.268	0.75 (0.45–1.25)
Controls			0.019	0/35/901	35/1837		
3_6901914	6901914						
Cases		C/CTCTT	0.022	1/39/885	41/1809	0.766	1.07 (0.69–1.67)
Controls			0.021	0/38/878	38/1794		
rs62237228	6902167						
Cases		C/A	0.190	30/290/601	350/1492	0.228	1.11 (0.94–1.31)
Controls			0.175	27/267/625	321/1517		
rs342034	6903601						
Cases		A/G	0.040	4/64/837	72/1738	0.351	1.18 (0.83–1.67)
Controls			0.034	1/60/852	62/1764		
rs35106713	7188180						
Cases		C/T	0.008	0/14/915	14/1844	0.188	0.64 (0.33–1.25)
Controls			0.012	0/22/914	22/1850		
rs140139253	7188396						
Cases		T/C	0.004	0/7/927	7/1861	0.787	1.16 (0.39–3.47)
Controls			0.003	0/6/924	6/1854		
GRM7_3f_7313045	7338045						
Cases		G/A	0.001	0/2/927	2/1856	0.157	NA
Controls			0.000	0/0/932	0/1864		
rs138571076	7338986						
Cases		G/A	0.002	0/3/926	3/1855	0.083	NA
Controls			0.000	0/0/932	0/1864		
rs192193072	7339471						
Cases		A/G	0.001	0/2/926	2/1854	0.156	0.33 (0.07–1.65)
Controls			0.003	0/6/919	6/1844		
rs712774	7456675						
Cases		C/T	0.263	81/392/580	554/1552	0.853	0.99 (0.86–1.14)
Controls			0.266	65/353/491	483/1335		
GRM7_nPb_7467774	7492774						
Cases		T/C	0.001	0/1/923	1/1847	0.321	NA
Controls			0.000	0/0/909	0/1818		
rs2229902	7494417						
Cases		T/A	0.447	185/445/282	815/1009	0.227	0.92 (0.81–1.05)
Controls			0.467	213/442/275	868/992		
rs1965222	7603194						
Cases		T/C	0.128	18/235/805	271/1845	0.713	1.04 (0.86–1.25)
Controls			0.124	11/210/713	232/1636		
GRM7_9c_7698252	7723252						
Cases		A/G	0.000	0/0/924	0/1848	0.319	NA
Controls			0.001	0/1/928	1/1857		
rs140995942	7723896						
Cases		A/G	0.013	0/24/909	24/1842	0.371	0.78 (0.46–1.34)
Controls			0.016	0/30/885	30/1800		
rs56173829	7782494						
Cases		A/T	0.007	0/12/899	12/1810	**0.035**	0.48 (0.24–0.96)
Controls			0.014	0/25/898	25/1821		
rs17726576	7782551						
Cases		T/C	0.027	1/47/874	49/1795	0.763	1.07 (0.71–1.6)
Controls			0.025	0/46/874	46/1794		
rs150288969	7783347						
Cases		A/G	0.008	0/14/910	14/1834	0.682	1.18 (0.54–2.55)
Controls			0.006	0/12/918	12/1848		

Min, minor; Maj, major; MAF, minor allele frequency; OR, odds ratio; CI, confidence interval.

*P* values, 0.05 need to be in bold.

Additionally, three SNPs were present only in cases, rs138571076 (*P* = 0.0826) in three, GRM7_3f_7313045 in two and GRM7_nPb_7467774 in only one bipolar patient. One of the novel SNPs, GRM7_9c_7698252 was found in only one control. All these SNPs are intronic in the *GRM7* gene. The SNP rs712774 was predicted to introduce a splice acceptor site using the Splice Site Prediction by Neural Network (http://www.fruitfly.org/seq_tools/splice.html) but was not associated with BP in our sample (*P* = 0.853). Imputation using European sample data from 1000 genomes did not reveal any synonymous or functional SNPs that were associated with BP (Supplementary Table SVI). A gene burden test did not provide any evidence of association with BD (C-alpha *P*-value = 0.233).

### Haplotype Analysis

We performed haplotype analysis on the combined data from all the SNPs genotyped in *GRM7* (UCL1 GWAS SNPs, the three GWAS SNPs in UCL2, and SNPs found by mutation screening in *GRM7*) in the present study using Haploview [Barrett et al., 2005]. The most significant haplotype (HAP_GRM7) associated with BP comprising SNPs rs1400166, rs2875257, rs10510353, rs11708019, rs1963265, rs1508724, rs9823996, rs11710946, and rs6769814 (*P* = 0.007) was less common in cases compared to controls (2.2% and 4.3% in cases and controls, respectively) (data not shown). Particularly, the SNPs driving the association of this haplotype were the three most significantly associated SNPs in UCL1 GWAS, rs1508724, rs11710946, and rs6769814. All the other associated haplotypes, including HAP_GRM7, did not survive permutation testing (data not shown).

### CNV Validation

**Figure 1:**
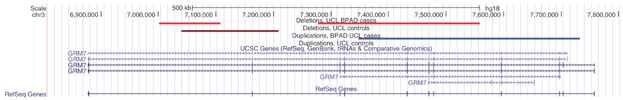
CNVs occurring in GRM7 in UCL1 GWAS sample [McQuillin et al., 2011].

McQuillin et al. [2011] reported an analysis of copy number variants in the UCL1 bipolar research sample and found that the overall rate of CNVs was significantly lower in cases compared to controls. Both deletions and duplications of size >100 kb were detected in *GRM7* in the UCL1 sample (Supplementary Table SVII). Deletions were present in two bipolar samples and one control sample and a duplication was found in only one case sample (Fig. 1).

We selected a deletion and a duplication involving a common genomic region for validation using QRT-PCR assay and TaqMan® RNase P CNR Assay, Human (Applied Biosystems). In the RNase P CNR assay, four individuals, two with a deletion, one with duplication, and two CNV negative control individuals were tested using primer pairs designed to detect duplication and deletion in the same assay. Replicable results were obtained with all the four primer pairs tested and the CNVs were confirmed in the respective UCL samples (Supplementary Fig. S1).

## DISCUSSION

The *GRM7* SNP markers found to be associated with BP in the UCL1 sample could not be replicated in the UCL2 sample. This can be explained by the presence of heterogeneity even within a single ancestral (UK and Irish) group of people affected by BP. The presence and the proportion of several other disease haplotypes in *GRM7* increasing susceptibility to BP in different populations may make it difficult to replicate findings even if the effect of *GRM7* is widespread. Another limitation may be that the replication sample was not large enough to detect the expected level of association with *GRM7*. Nonetheless, two of the previously associated SNPs, rs1508724 and rs6769814, in UCL1 remained significant when we combined genotype data of the UCL1 and UCL2 samples using PLINK. Resequencing of *GRM7* in the selected bipolar cases detected a 3′-UTR variant, rs56173829, which was significantly associated with BP. This variant was less common in cases compared to controls. Variants in the 3′-UTR play an important role in the regulation of translation by means of miRNA binding sites. Additionally, we found two novel singleton mutations (GRM7_3f_7313045, GRM7_nPb_7467774) and one previously published SNP (rs138571076) that were only present in cases and not observed in control volunteers. We used bi-directional C-alpha test rare variant aggregate approach to test for gene-level association between bipolar cases and controls. Although we did not find any evidence of a greater burden of the rare variants in *GRM7* among bipolar cases compared to controls in our study it might be due to the small sample size considering only 32 bipolar samples were sequenced in our study.

Alternative splicing in *GRM7* results in five isoforms characterized by different C-terminals. The rs56173829 variant is located in the 3′-UTR of the long isoform of *GRM7* (NM_181874; ENST00000486284). RNA webserver was used to predict the secondary structure of the wild type and mutant 3′-UTR containing SNP rs56173829. The RNA webserver produced two structures—minimum free energy (MFE) and centroid secondary structure. The MFE structure of an RNA sequence is the secondary structure that contributes a minimum of free energy whereas the centroid structure of the same is the secondary structure with minimal base pair distance to all other secondary structures in the Boltzmann ensemble. The predicted centroid secondary structures for the wild-type 3′-UTR was altered by the introduction of the SNP rs56173829, however, the MFE structures for both were not dissimilar (Supplementary Figs. S2 and S3). The *cis*-regulatory elements in the 3′-UTR of mRNAs have been reported to influence translation and cause diseases. Translation de-regulation and disorders resulting from mutations affecting the termination codon, polyadenylation signal, and secondary structure of 3′-UTR of mRNA have been documented [Chatterjee and Pal, 2009]. A correlation between the functionality of 3′-UTR variants and alterations in the predicted mRNA secondary structure was reported in a study by Chen et al. [2006] on 83 disease associated 3′-UTR variants of various human mRNAs.

miRNAs are 21–22 nucleotides long, single-stranded, and bind to 3′-UTRs of particular mRNAs through partially complementary sequences and prevents the mRNAs from being translated into proteins. Multiple types of miRNAs can cooperate to suppress translation of a single mRNA, and a single miRNA can interact with multiple types of miRNAs, thereby regulating the protein expression of different genes [Filipowicz et al., 2008]. RegRNA (http://regrna.mbc.nctu.edu.tw/html/prediction.html) is a web server that facilitates the analysis of regulatory RNA motifs. The software predicted differential miRNA binding of the wild type and mutant 3′-UTR containing the rs56173829 base. Target scan and the UCSC genome browser do not predict any miRNA binding sites involving the nucleotide and the sequence around it. In a study by Zhou et al. [2009] chronic treatment of primary cultures with mood stabilizers, VPA or lithium elevated levels of GRM7 and lowered levels of miR-34a. Incubation of primary hippocampal culture with miR-34a precursor significantly reduced GRM7 protein levels whereas, with anti-miR miRNA-34a inhibitor, significantly increased GRM7 protein levels were observed. These results suggested that mood stabilizers produce a part of their behavioral effects through mechanisms including modulation of protein levels through miRNAs such as miR-34a [Zhou et al., 2009].

CNVs have been implicated in the pathogenesis of many psychiatric disorders however CNVs associated with BP have been difficult to find [Zhang et al., 2009; Grozeva et al., 2010; McQuillin et al., 2011]. We confirmed the presence of two deletions and a duplication in the UCL1 sample. Functional studies investigating the possible role of these CNVs are required to shed more light on their involvement in the disorder.

In conclusion, we found a 3′-UTR variant in *GRM7* that was significantly associated with BP. Bioinformatic analyses predicted that the rs56173829 variant influenced the centroid secondary structure of the RNA and also altered the binding site of a few miRNAs. However, results from these in silico analyses should be interpreted with caution as these are based on mathematical algorithms instead of expression studies. Thus, functional assays need to be carried out to assess the role of the 3′-UTR variant on *GRM7* gene expression. The case-only novel mutations reported in our study also warrant further investigation in other populations in order to understand their distribution and involvement in the etiology of BP.
