# Supplementary material for: Allelic Association, DNA Resequencing and Copy Number Variation at the Metabotropic Glutamate Receptor GRM7 Gene Locus in Bipolar Disorder
Source: Am J Med Genet B Neuropsychiatr Genet. 2014 May 8;165(4):365–72. doi: 10.1002/ajmg.b.32239 (PMC4231221; doi:10.1002/ajmg.b.32239)
Supplement: Supplementary file 1 — Supporting Information. [file ajmg0165-0365-SD1.doc]

**Supplementary figure I. CNV validation using TaqMan® RNase P copy number reference assay**

**
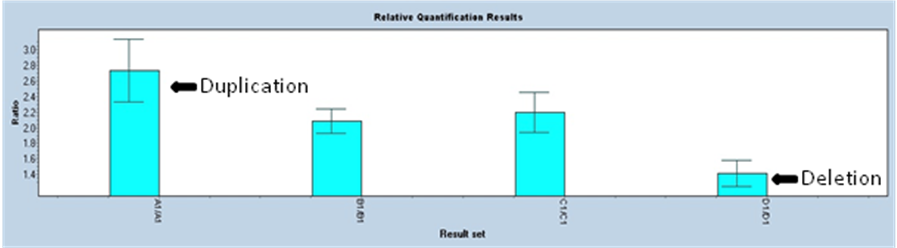
**

(a)

(b)

**Supplementary figure II. Centroid secondary structure for the wild-type (a) and mutant (b) 5' UTR containing SNP rs56173829 predicted using RNAfold webserver.**

(a)

(b)

**Supplementary figure III. MFE secondary structure for the wild-type (a) and mutant (b) 5' UTR containing SNP rs56173829 predicted using RNAfold webserver.**

**Supplementary table S1: Association of *GRM7* SNPs in the UCL1 sample (SNP data from our bipolar GWAS (Sklar et al., 2008))**.

| **SNP ID** | **BP** | **A1** | **F_A** | **F_U** | **A2** | **CHISQ** | **P** | **OR** | **SE** | **L95** | **U95** |
| --- | --- | --- | --- | --- | --- | --- | --- | --- | --- | --- | --- |
| rs1400286 | 6861715 | A | 0.04144 | 0.04813 | C | 0.5522 | 0.4574 | 0.8548 | 0.2112 | 0.565 | 1.293 |
| rs17046124 | 6861866 | C | 0.01287 | 0.005882 | T | 2.733 | 0.09827 | 2.203 | 0.4899 | 0.8433 | 5.755 |
| rs17288442 | 6866379 | A | 0.2965 | 0.3323 | C | 3.129 | 0.0769 | 0.8467 | 0.09412 | 0.7041 | 1.018 |
| rs17046139 | 6869166 | T | 0.06434 | 0.07059 | C | 0.3273 | 0.5672 | 0.9054 | 0.1738 | 0.644 | 1.273 |
| rs342045 | 6885895 | C | 0.1232 | 0.1363 | T | 0.8029 | 0.3702 | 0.8903 | 0.1298 | 0.6903 | 1.148 |
| rs342040 | 6891874 | C | 0.1185 | 0.133 | T | 0.9953 | 0.3184 | 0.8761 | 0.1327 | 0.6755 | 1.136 |
| rs3749380 | 6903297 | T | 0.3833 | 0.3961 | C | 0.3631 | 0.5468 | 0.9476 | 0.08937 | 0.7953 | 1.129 |
| rs340659 | 6906622 | C | 0.06814 | 0.04804 | T | 3.864 | 0.04932 | 1.449 | 0.1896 | 0.9993 | 2.101 |
| rs340657 | 6907600 | G | 0.443 | 0.4314 | A | 0.29 | 0.5902 | 1.048 | 0.08787 | 0.8826 | 1.246 |
| rs456835 | 6911400 | T | 0.414 | 0.419 | C | 0.05218 | 0.8193 | 0.9799 | 0.08871 | 0.8236 | 1.166 |
| rs17046231 | 6913374 | T | 0.05882 | 0.06961 | C | 1.021 | 0.3122 | 0.8354 | 0.1782 | 0.5892 | 1.185 |
| rs9870018 | 6927311 | T | 0.2523 | 0.2682 | C | 0.6859 | 0.4075 | 0.9209 | 0.09952 | 0.7577 | 1.119 |
| rs3846161 | 6939834 | G | 0.2151 | 0.2348 | A | 1.172 | 0.279 | 0.8931 | 0.1045 | 0.7277 | 1.096 |
| rs11915789 | 6950603 | A | 0.02091 | 0.02357 | T | 0.1642 | 0.6853 | 0.885 | 0.3016 | 0.4901 | 1.598 |
| rs17046322 | 6952739 | A | 0.04604 | 0.03543 | G | 1.505 | 0.2199 | 1.314 | 0.2231 | 0.8485 | 2.034 |
| rs10490857 | 6959982 | G | 0.1411 | 0.1441 | A | 0.03801 | 0.8454 | 0.976 | 0.1247 | 0.7643 | 1.246 |
| SNP_A-4194743 | 6962448 | C | 0.02033 | 0.01287 | T | 1.768 | 0.1836 | 1.592 | 0.3526 | 0.7975 | 3.177 |
| rs2116711 | 6968207 | T | 0.09982 | 0.1048 | C | 0.1403 | 0.7079 | 0.9473 | 0.1447 | 0.7134 | 1.258 |
| rs17694650 | 7001308 | T | 0.1112 | 0.1125 | C | 0.00929 | 0.9232 | 0.9867 | 0.1394 | 0.7508 | 1.297 |
| rs4686101 | 7006880 | C | 0.4575 | 0.4555 | T | 0.008035 | 0.9286 | 1.008 | 0.08779 | 0.8486 | 1.197 |
| rs4686102 | 7006960 | T | 0.02354 | 0.02705 | C | 0.2581 | 0.6114 | 0.867 | 0.2811 | 0.4997 | 1.504 |
| rs17751439 | 7010587 | G | 0.01845 | 0.01775 | A | 0.01438 | 0.9045 | 1.04 | 0.3279 | 0.547 | 1.978 |
| rs781393 | 7011534 | C | 0.2519 | 0.245 | T | 0.1316 | 0.7168 | 1.038 | 0.1021 | 0.8496 | 1.268 |
| rs1356268 | 7021095 | G | 0.04638 | 0.04314 | A | 0.1289 | 0.7195 | 1.079 | 0.2115 | 0.7128 | 1.633 |
| rs6801970 | 7026273 | C | 0.295 | 0.2922 | G | 0.02105 | 0.8846 | 1.014 | 0.09571 | 0.8406 | 1.223 |
| rs576913 | 7026696 | A | 0.1388 | 0.1598 | G | 1.834 | 0.1756 | 0.8473 | 0.1224 | 0.6665 | 1.077 |
| rs6808554 | 7028319 | A | 0.08656 | 0.101 | G | 1.291 | 0.2559 | 0.8436 | 0.1498 | 0.629 | 1.132 |
| rs2069062 | 7034549 | C | 0.1934 | 0.2078 | G | 0.6876 | 0.407 | 0.9137 | 0.1089 | 0.7381 | 1.131 |
| rs1499161 | 7045455 | C | 0.4724 | 0.4941 | A | 0.9957 | 0.3183 | 0.9166 | 0.08727 | 0.7725 | 1.088 |
| SNP_A-1937262 | 7046445 | C | 0.1971 | 0.2126 | G | 0.7795 | 0.3773 | 0.9089 | 0.1082 | 0.7353 | 1.124 |
| rs1532544 | 7052752 | A | 0.1679 | 0.1755 | G | 0.2032 | 0.6521 | 0.9482 | 0.118 | 0.7524 | 1.195 |
| rs6781223 | 7065496 | C | 0.464 | 0.4863 | T | 1.044 | 0.307 | 0.9146 | 0.08737 | 0.7707 | 1.085 |
| rs11711367 | 7077524 | T | 0.4216 | 0.4436 | A | 1.018 | 0.3129 | 0.9145 | 0.08853 | 0.7689 | 1.088 |
| rs6771606 | 7085713 | G | 0.2072 | 0.1831 | A | 1.94 | 0.1637 | 1.166 | 0.1104 | 0.9392 | 1.448 |
| rs17234886 | 7092021 | C | 0.2177 | 0.2366 | G | 1.059 | 0.3034 | 0.898 | 0.1045 | 0.7317 | 1.102 |
| rs9820417 | 7104786 | G | 0.2303 | 0.2672 | A | 3.645 | 0.05622 | 0.8206 | 0.1037 | 0.6697 | 1.005 |
| rs17824866 | 7104822 | C | 0.2255 | 0.2475 | T | 1.392 | 0.2381 | 0.8853 | 0.1033 | 0.723 | 1.084 |
| rs6803027 | 7110070 | A | 0.05515 | 0.05906 | G | 0.1492 | 0.6993 | 0.93 | 0.188 | 0.6433 | 1.344 |
| rs6777970 | 7110108 | A | 0.05525 | 0.06004 | G | 0.2221 | 0.6375 | 0.9155 | 0.1873 | 0.6342 | 1.322 |
| rs6772333 | 7116696 | G | 0.2677 | 0.2959 | A | 2.04 | 0.1532 | 0.8701 | 0.09743 | 0.7189 | 1.053 |
| rs9311976 | 7122638 | A | 0.2421 | 0.2292 | G | 0.4824 | 0.4873 | 1.074 | 0.1031 | 0.8777 | 1.315 |
| rs9812630 | 7124872 | A | 0.4006 | 0.4069 | G | 0.08701 | 0.768 | 0.9741 | 0.08887 | 0.8184 | 1.159 |
| rs9875041 | 7125230 | C | 0.4061 | 0.4157 | A | 0.194 | 0.6596 | 0.9614 | 0.08937 | 0.8069 | 1.145 |
| rs9856068 | 7125486 | A | 0.05268 | 0.05588 | C | 0.105 | 0.746 | 0.9395 | 0.1926 | 0.6441 | 1.37 |
| rs6785425 | 7125901 | C | 0.05 | 0.05413 | T | 0.1815 | 0.6701 | 0.9196 | 0.1968 | 0.6253 | 1.352 |
| rs13072518 | 7131501 | T | 0.4476 | 0.4588 | G | 0.2671 | 0.6053 | 0.9558 | 0.08755 | 0.805 | 1.135 |
| rs9826341 | 7132442 | C | 0.414 | 0.4239 | G | 0.2091 | 0.6475 | 0.9603 | 0.08863 | 0.8072 | 1.142 |
| SNP_A-2134151 | 7138976 | G | 0.05147 | 0.05392 | A | 0.0634 | 0.8012 | 0.9521 | 0.195 | 0.6496 | 1.395 |
| rs1909397 | 7139094 | A | 0.4372 | 0.4518 | T | 0.4535 | 0.5007 | 0.9425 | 0.08792 | 0.7933 | 1.12 |
| rs9837834 | 7142227 | G | 0.3814 | 0.39 | A | 0.1622 | 0.6872 | 0.9646 | 0.08958 | 0.8093 | 1.15 |
| rs9837989 | 7142307 | C | 0.3801 | 0.3907 | T | 0.2441 | 0.6213 | 0.9565 | 0.09011 | 0.8016 | 1.141 |
| rs6764411 | 7143551 | C | 0.3824 | 0.3927 | A | 0.2378 | 0.6258 | 0.9573 | 0.08955 | 0.8032 | 1.141 |
| rs9876724 | 7152640 | A | 0.3665 | 0.4008 | G | 2.616 | 0.1058 | 0.8649 | 0.08976 | 0.7254 | 1.031 |
| rs6777701 | 7153339 | C | 0.4456 | 0.4702 | T | 1.28 | 0.2578 | 0.9054 | 0.08784 | 0.7622 | 1.075 |
| rs6778030 | 7153652 | C | 0.461 | 0.4862 | T | 1.335 | 0.248 | 0.9038 | 0.08755 | 0.7613 | 1.073 |
| rs11928865 | 7155702 | A | 0.2532 | 0.2701 | T | 0.7786 | 0.3776 | 0.9162 | 0.09926 | 0.7542 | 1.113 |
| rs6443090 | 7168009 | G | 0.1875 | 0.1873 | C | 0.000208 | 0.9885 | 1.002 | 0.1117 | 0.8047 | 1.247 |
| rs9814809 | 7170741 | C | 0.2546 | 0.2761 | G | 1.237 | 0.266 | 0.8956 | 0.09915 | 0.7374 | 1.088 |
| rs1878164 | 7173002 | T | 0.4328 | 0.4559 | C | 1.137 | 0.2863 | 0.9107 | 0.08777 | 0.7667 | 1.082 |
| rs4441639 | 7179682 | T | 0.2569 | 0.2805 | C | 1.484 | 0.2232 | 0.8869 | 0.09858 | 0.7311 | 1.076 |
| rs3828472 | 7188006 | G | 0.2994 | 0.3051 | T | 0.07996 | 0.7774 | 0.9735 | 0.09512 | 0.8079 | 1.173 |
| rs3749448 | 7188116 | T | 0.1957 | 0.2006 | C | 0.07762 | 0.7806 | 0.9699 | 0.1098 | 0.7821 | 1.203 |
| rs7650218 | 7191923 | A | 0.1951 | 0.1944 | G | 0.001767 | 0.9665 | 1.005 | 0.1112 | 0.8079 | 1.249 |
| rs9814881 | 7198602 | G | 0.1895 | 0.1905 | A | 0.003252 | 0.9545 | 0.9936 | 0.112 | 0.7978 | 1.238 |
| rs12497688 | 7201316 | C | 0.1859 | 0.1887 | T | 0.02806 | 0.867 | 0.9814 | 0.1122 | 0.7876 | 1.223 |
| rs17235018 | 7212512 | G | 0.3208 | 0.3327 | C | 0.3386 | 0.5606 | 0.9473 | 0.09302 | 0.7894 | 1.137 |
| rs17288561 | 7212607 | T | 0.3686 | 0.3703 | C | 0.007056 | 0.9331 | 0.9924 | 0.09034 | 0.8314 | 1.185 |
| rs11131064 | 7214945 | T | 0.3787 | 0.3477 | C | 2.175 | 0.1403 | 1.143 | 0.09076 | 0.9569 | 1.366 |
| rs1400166 | 7218175 | T | 0.3056 | 0.321 | C | 0.5695 | 0.4505 | 0.9309 | 0.09483 | 0.773 | 1.121 |
| rs2875257 | 7221090 | A | 0.3088 | 0.3353 | G | 1.691 | 0.1935 | 0.8858 | 0.0933 | 0.7377 | 1.064 |
| rs10510353 | 7221165 | C | 0.3051 | 0.3343 | T | 2.061 | 0.1511 | 0.8744 | 0.09349 | 0.728 | 1.05 |
| rs11708019 | 7229619 | G | 0.3327 | 0.3755 | A | 4.214 | 0.04009 | 0.8293 | 0.09122 | 0.6935 | 0.9916 |
| rs1963265 | 7233935 | T | 0.3404 | 0.3089 | G | 2.363 | 0.1242 | 1.155 | 0.09352 | 0.9612 | 1.387 |
| rs1508724 | 7241745 | A | 0.3346 | 0.2745 | G | 8.947 | 0.002779 | 1.329 | 0.09514 | 1.103 | 1.601 |
| rs9823996 | 7244509 | G | 0.329 | 0.2863 | C | 4.515 | 0.03359 | 1.223 | 0.09466 | 1.016 | 1.472 |
| rs11710946 | 7246241 | A | 0.3603 | 0.4216 | G | 8.309 | 0.003945 | 0.7728 | 0.08949 | 0.6485 | 0.9209 |
| rs6769814 | 7251433 | G | 0.3401 | 0.2917 | A | 5.669 | 0.01726 | 1.251 | 0.09419 | 1.04 | 1.505 |
| rs10510354 | 7252190 | C | 0.3631 | 0.4216 | G | 7.568 | 0.005941 | 0.7821 | 0.08942 | 0.6564 | 0.9319 |
| rs13070476 | 7259313 | C | 0.2197 | 0.254 | T | 3.376 | 0.06616 | 0.8273 | 0.1033 | 0.6757 | 1.013 |
| rs951557 | 7269035 | T | 0.443 | 0.4451 | C | 0.009256 | 0.9234 | 0.9916 | 0.08772 | 0.835 | 1.178 |
| rs2136152 | 7282402 | T | 0.1562 | 0.1745 | G | 1.274 | 0.2591 | 0.876 | 0.1174 | 0.696 | 1.103 |
| rs4686119 | 7282739 | T | 0.1645 | 0.1804 | C | 0.9302 | 0.3348 | 0.8947 | 0.1154 | 0.7136 | 1.122 |
| rs908465 | 7283715 | T | 0.1633 | 0.1812 | G | 1.177 | 0.2781 | 0.8818 | 0.116 | 0.7024 | 1.107 |
| rs1605705 | 7290044 | G | 0.1562 | 0.1725 | A | 1.019 | 0.3127 | 0.888 | 0.1176 | 0.7052 | 1.118 |
| rs4095095 | 7309658 | T | 0.3139 | 0.3139 | C | 1.83E-06 | 0.9989 | 1 | 0.09432 | 0.8313 | 1.203 |
| rs6443099 | 7320514 | C | 0.4006 | 0.4059 | T | 0.0621 | 0.8032 | 0.9781 | 0.08889 | 0.8217 | 1.164 |
| rs1876614 | 7321289 | A | 0.1596 | 0.1729 | G | 0.6701 | 0.413 | 0.9085 | 0.1172 | 0.722 | 1.143 |
| SNP_A-4250891 | 7321712 | G | 0.009242 | 0.00789 | A | 0.1125 | 0.7373 | 1.173 | 0.4764 | 0.4611 | 2.984 |
| rs12637466 | 7321909 | T | 0.4292 | 0.4127 | C | 0.5867 | 0.4437 | 1.07 | 0.0883 | 0.8999 | 1.272 |
| rs7635212 | 7328063 | G | 0.3803 | 0.3931 | A | 0.3659 | 0.5453 | 0.9473 | 0.08953 | 0.7948 | 1.129 |
| rs6443100 | 7333382 | T | 0.3759 | 0.3947 | G | 0.7814 | 0.3767 | 0.9238 | 0.08965 | 0.7749 | 1.101 |
| rs7632044 | 7335947 | C | 0.1621 | 0.1722 | G | 0.3912 | 0.5317 | 0.9295 | 0.117 | 0.739 | 1.169 |
| rs2133450 | 7336452 | C | 0.4524 | 0.42 | A | 2.211 | 0.137 | 1.141 | 0.08872 | 0.9589 | 1.358 |
| rs2291867 | 7340164 | G | 0.3696 | 0.3894 | A | 0.8638 | 0.3527 | 0.9194 | 0.09047 | 0.77 | 1.098 |
| SNP_A-1971589 | 7341024 | G | 0.4594 | 0.4222 | A | 2.938 | 0.08652 | 1.163 | 0.08803 | 0.9785 | 1.382 |
| SNP_A-1971590 | 7341994 | A | 0.3759 | 0.399 | G | 1.184 | 0.2765 | 0.9072 | 0.08948 | 0.7613 | 1.081 |
| rs17047073 | 7349612 | A | 0.381 | 0.3996 | G | 0.7635 | 0.3822 | 0.9248 | 0.08952 | 0.7759 | 1.102 |
| rs9849147 | 7350358 | A | 0.3704 | 0.3902 | T | 0.8753 | 0.3495 | 0.9194 | 0.08979 | 0.7711 | 1.096 |
| rs10510356 | 7350877 | C | 0.3824 | 0.3988 | G | 0.5994 | 0.4388 | 0.9331 | 0.08938 | 0.7832 | 1.112 |
| rs17697853 | 7366195 | A | 0.4375 | 0.402 | G | 2.729 | 0.09854 | 1.157 | 0.08839 | 0.9731 | 1.376 |
| rs9990013 | 7366352 | A | 0.4384 | 0.4006 | C | 3.086 | 0.07897 | 1.168 | 0.0885 | 0.9821 | 1.389 |
| rs7621537 | 7372535 | C | 0.3419 | 0.3745 | G | 2.435 | 0.1187 | 0.8677 | 0.09094 | 0.7261 | 1.037 |
| rs17047149 | 7380288 | G | 0.005515 | 0.01282 | A | 3.127 | 0.07698 | 0.427 | 0.4955 | 0.1617 | 1.128 |
| rs10510364 | 7381271 | T | 0.1155 | 0.1258 | C | 0.5008 | 0.4792 | 0.9079 | 0.1365 | 0.6947 | 1.187 |
| rs6810141 | 7383890 | A | 0.4314 | 0.4266 | G | 0.04832 | 0.826 | 1.02 | 0.08853 | 0.8572 | 1.213 |
| rs1508720 | 7384636 | A | 0.1206 | 0.122 | C | 0.009945 | 0.9206 | 0.9868 | 0.1337 | 0.7593 | 1.282 |
| rs1499199 | 7386444 | C | 0.4072 | 0.3951 | A | 0.3193 | 0.572 | 1.052 | 0.08894 | 0.8833 | 1.252 |
| rs11131069 | 7386487 | G | 0.1654 | 0.1798 | C | 0.7608 | 0.3831 | 0.904 | 0.1157 | 0.7205 | 1.134 |
| rs9836538 | 7387400 | G | 0.3969 | 0.3842 | C | 0.3552 | 0.5512 | 1.055 | 0.08962 | 0.8849 | 1.257 |
| rs9837019 | 7387710 | T | 0.3971 | 0.3843 | C | 0.3592 | 0.549 | 1.055 | 0.08935 | 0.8855 | 1.257 |
| rs17047183 | 7389204 | T | 0.09444 | 0.1069 | C | 0.8951 | 0.3441 | 0.8717 | 0.1453 | 0.6557 | 1.159 |
| rs989126 | 7389526 | C | 0.3137 | 0.3089 | A | 0.05339 | 0.8173 | 1.022 | 0.09579 | 0.8474 | 1.234 |
| rs7628504 | 7390577 | G | 0.2143 | 0.1962 | A | 1.038 | 0.3082 | 1.117 | 0.109 | 0.9025 | 1.384 |
| rs17047199 | 7392719 | T | 0.09375 | 0.1088 | A | 1.317 | 0.2512 | 0.8472 | 0.1447 | 0.638 | 1.125 |
| rs7622749 | 7397946 | T | 0.1232 | 0.1375 | C | 0.9586 | 0.3275 | 0.8809 | 0.1296 | 0.6833 | 1.136 |
| rs7340751 | 7414354 | G | 0.4705 | 0.4706 | T | 6.19E-06 | 0.998 | 0.9998 | 0.08736 | 0.8425 | 1.186 |
| rs2324122 | 7414789 | G | 0.4162 | 0.4081 | A | 0.142 | 0.7063 | 1.034 | 0.08877 | 0.8689 | 1.231 |
| rs10510366 | 7415452 | T | 0.1447 | 0.1591 | C | 0.8463 | 0.3576 | 0.894 | 0.1218 | 0.7042 | 1.135 |
| rs1499079 | 7433728 | A | 0.05463 | 0.04635 | C | 0.7458 | 0.3878 | 1.189 | 0.2006 | 0.8024 | 1.762 |
| rs1499204 | 7437037 | C | 0.4553 | 0.4802 | G | 1.301 | 0.2541 | 0.9047 | 0.08782 | 0.7616 | 1.075 |
| rs12489041 | 7437599 | G | 0.4594 | 0.4882 | A | 1.752 | 0.1857 | 0.8908 | 0.0874 | 0.7506 | 1.057 |
| rs712767 | 7443688 | T | 0.3854 | 0.3703 | C | 0.5061 | 0.4768 | 1.066 | 0.09008 | 0.8936 | 1.272 |
| rs1066658 | 7445630 | G | 0.384 | 0.3696 | A | 0.4651 | 0.4953 | 1.063 | 0.09016 | 0.8912 | 1.269 |
| rs6775424 | 7448339 | A | 0.5138 | 0.4833 | G | 1.953 | 0.1623 | 1.13 | 0.08721 | 0.9521 | 1.34 |
| rs712775 | 7458710 | G | 0.2486 | 0.248 | A | 0.000951 | 0.9754 | 1.003 | 0.1011 | 0.8228 | 1.223 |
| rs712777 | 7463958 | C | 0.2518 | 0.2436 | T | 0.1915 | 0.6616 | 1.045 | 0.1011 | 0.8573 | 1.274 |
| rs11717750 | 7464013 | T | 0.5 | 0.4617 | C | 3.089 | 0.07882 | 1.166 | 0.08738 | 0.9824 | 1.384 |
| rs17655560 | 7466155 | G | 0.4559 | 0.4863 | C | 1.952 | 0.1624 | 0.8851 | 0.08735 | 0.7459 | 1.05 |
| rs712779 | 7476597 | T | 0.1575 | 0.1412 | C | 1.096 | 0.2951 | 1.137 | 0.1226 | 0.8941 | 1.446 |
| rs712782 | 7481801 | G | 0.1593 | 0.1402 | A | 1.505 | 0.2199 | 1.162 | 0.1225 | 0.914 | 1.477 |
| rs17717959 | 7481854 | T | 0.5084 | 0.4743 | C | 2.42 | 0.1198 | 1.146 | 0.08772 | 0.9651 | 1.361 |
| rs712785 | 7485714 | C | 0.1507 | 0.1343 | G | 1.16 | 0.2815 | 1.144 | 0.1249 | 0.8955 | 1.461 |
| rs779701 | 7518772 | G | 0.2902 | 0.3078 | A | 0.7798 | 0.3772 | 0.9193 | 0.09533 | 0.7626 | 1.108 |
| rs779699 | 7519647 | C | 0.2745 | 0.2967 | G | 1.264 | 0.2608 | 0.897 | 0.09669 | 0.7421 | 1.084 |
| rs752300 | 7520084 | T | 0.2677 | 0.296 | C | 2.062 | 0.151 | 0.8694 | 0.09751 | 0.7181 | 1.052 |
| rs779694 | 7521741 | G | 0.2878 | 0.3084 | C | 1.068 | 0.3014 | 0.9061 | 0.09542 | 0.7515 | 1.092 |
| rs1083801 | 7523498 | G | 0.2831 | 0.2588 | A | 1.568 | 0.2105 | 1.131 | 0.09818 | 0.9328 | 1.371 |
| rs779706 | 7524042 | G | 0.2895 | 0.3098 | C | 1.033 | 0.3096 | 0.9079 | 0.09515 | 0.7534 | 1.094 |
| rs779705 | 7524103 | G | 0.2831 | 0.3078 | A | 1.551 | 0.213 | 0.8878 | 0.09555 | 0.7362 | 1.071 |
| rs779733 | 7527372 | A | 0.2405 | 0.2454 | C | 0.06846 | 0.7936 | 0.9733 | 0.1034 | 0.7947 | 1.192 |
| rs9870680 | 7529555 | A | 0.4596 | 0.4569 | G | 0.01541 | 0.9012 | 1.011 | 0.08747 | 0.8516 | 1.2 |
| rs12494654 | 7533393 | T | 0.4329 | 0.4382 | C | 0.06086 | 0.8051 | 0.9785 | 0.0879 | 0.8237 | 1.163 |
| rs10222587 | 7533618 | T | 0.1271 | 0.1257 | C | 0.008426 | 0.9269 | 1.012 | 0.1321 | 0.7814 | 1.311 |
| rs3804945 | 7539974 | T | 0.3505 | 0.3517 | C | 0.003312 | 0.9541 | 0.9947 | 0.09173 | 0.8311 | 1.191 |
| rs11131078 | 7548067 | T | 0.3217 | 0.3127 | C | 0.1945 | 0.6592 | 1.042 | 0.09367 | 0.8674 | 1.252 |
| rs756084 | 7561149 | C | 0.454 | 0.4283 | A | 1.404 | 0.236 | 1.11 | 0.0879 | 0.9341 | 1.318 |
| rs1106486 | 7562364 | C | 0.3336 | 0.35 | T | 0.6247 | 0.4293 | 0.9299 | 0.09201 | 0.7764 | 1.114 |
| rs1121606 | 7564242 | G | 0.4677 | 0.4454 | A | 1.038 | 0.3083 | 1.094 | 0.08791 | 0.9206 | 1.299 |
| rs9819987 | 7567664 | G | 0.4678 | 0.4461 | T | 1.004 | 0.3164 | 1.092 | 0.08752 | 0.9196 | 1.296 |
| rs3804928 | 7576514 | A | 0.203 | 0.2269 | G | 1.789 | 0.1811 | 0.8675 | 0.1063 | 0.7043 | 1.068 |
| rs779749 | 7576541 | T | 0.3633 | 0.3865 | C | 1.185 | 0.2764 | 0.9059 | 0.09082 | 0.7582 | 1.082 |
| rs779742 | 7583058 | G | 0.4733 | 0.4539 | A | 0.7939 | 0.3729 | 1.081 | 0.08746 | 0.9107 | 1.283 |
| rs17664833 | 7583314 | C | 0.09835 | 0.102 | G | 0.07639 | 0.7823 | 0.9607 | 0.1452 | 0.7228 | 1.277 |
| rs779741 | 7583602 | C | 0.4133 | 0.4333 | A | 0.8655 | 0.3522 | 0.9211 | 0.0883 | 0.7748 | 1.095 |
| rs1351938 | 7598417 | A | 0.4458 | 0.4637 | G | 0.6844 | 0.4081 | 0.9301 | 0.08754 | 0.7835 | 1.104 |
| rs17665113 | 7598811 | C | 0.1094 | 0.1061 | T | 0.059 | 0.8081 | 1.035 | 0.1407 | 0.7854 | 1.363 |
| rs1143740 | 7602077 | C | 0.3722 | 0.3833 | T | 0.2755 | 0.5996 | 0.9539 | 0.08989 | 0.7998 | 1.138 |
| rs1531939 | 7617025 | C | 0.2865 | 0.2958 | G | 0.2169 | 0.6414 | 0.9559 | 0.09676 | 0.7908 | 1.156 |
| rs1485174 | 7620828 | T | 0.2831 | 0.277 | C | 0.09623 | 0.7564 | 1.031 | 0.09713 | 0.8519 | 1.247 |
| rs1485172 | 7621158 | A | 0.4288 | 0.4283 | G | 0.000636 | 0.9799 | 1.002 | 0.08823 | 0.8431 | 1.191 |
| rs9826424 | 7621512 | A | 0.2887 | 0.2959 | G | 0.1282 | 0.7204 | 0.9662 | 0.09605 | 0.8004 | 1.166 |
| rs3804906 | 7621654 | A | 0.2877 | 0.2922 | C | 0.05117 | 0.821 | 0.9785 | 0.09605 | 0.8106 | 1.181 |
| rs3804904 | 7621977 | A | 0.286 | 0.2947 | C | 0.1937 | 0.6599 | 0.9586 | 0.09615 | 0.7939 | 1.157 |
| rs3792460 | 7622270 | C | 0.284 | 0.2879 | T | 0.03846 | 0.8445 | 0.981 | 0.09781 | 0.8099 | 1.188 |
| rs11716647 | 7624410 | A | 0.4256 | 0.4224 | G | 0.02143 | 0.8836 | 1.013 | 0.08824 | 0.8521 | 1.204 |
| rs11717471 | 7624469 | G | 0.2831 | 0.2804 | A | 0.01891 | 0.8906 | 1.013 | 0.09689 | 0.8381 | 1.225 |
| rs4143516 | 7624941 | C | 0.2848 | 0.2824 | T | 0.01413 | 0.9054 | 1.012 | 0.09736 | 0.8359 | 1.224 |
| rs1872400 | 7624974 | A | 0.427 | 0.4245 | G | 0.01318 | 0.9086 | 1.01 | 0.08826 | 0.8497 | 1.201 |
| rs3792457 | 7631222 | A | 0.08333 | 0.0789 | G | 0.1381 | 0.7102 | 1.061 | 0.1603 | 0.7752 | 1.453 |
| rs3804886 | 7636612 | C | 0.1939 | 0.2108 | G | 0.927 | 0.3356 | 0.9008 | 0.1085 | 0.7283 | 1.114 |
| rs3804883 | 7639708 | G | 0.2969 | 0.2971 | A | 8.52E-05 | 0.9926 | 0.9991 | 0.09538 | 0.8288 | 1.205 |
| rs11713266 | 7645217 | G | 0.1498 | 0.1451 | T | 0.09318 | 0.7602 | 1.038 | 0.123 | 0.8159 | 1.321 |
| rs1485171 | 7651185 | A | 0.1596 | 0.1542 | C | 0.1144 | 0.7352 | 1.041 | 0.12 | 0.8231 | 1.318 |
| rs17047734 | 7662963 | A | 0.07721 | 0.06287 | G | 1.655 | 0.1983 | 1.247 | 0.172 | 0.8903 | 1.747 |
| rs1485167 | 7664334 | C | 0.1765 | 0.1939 | T | 1.059 | 0.3035 | 0.8909 | 0.1123 | 0.7148 | 1.11 |
| rs6799329 | 7666694 | T | 0.4118 | 0.399 | C | 0.3547 | 0.5515 | 1.054 | 0.08879 | 0.8859 | 1.255 |
| rs3792452 | 7666784 | A | 0.1753 | 0.1931 | G | 1.117 | 0.2906 | 0.8879 | 0.1126 | 0.7121 | 1.107 |
| rs17723289 | 7666994 | G | 0.1685 | 0.1781 | C | 0.3408 | 0.5594 | 0.9349 | 0.1153 | 0.7458 | 1.172 |
| rs10510370 | 7667672 | T | 0.1682 | 0.1775 | A | 0.3155 | 0.5743 | 0.9373 | 0.1153 | 0.7478 | 1.175 |
| rs3804867 | 7667909 | T | 0.1682 | 0.1775 | C | 0.3155 | 0.5743 | 0.9373 | 0.1153 | 0.7478 | 1.175 |
| rs9860274 | 7673242 | T | 0.3833 | 0.3863 | G | 0.02005 | 0.8874 | 0.9874 | 0.08958 | 0.8284 | 1.177 |
| rs3804859 | 7678340 | C | 0.1471 | 0.1368 | A | 0.4526 | 0.5011 | 1.088 | 0.1251 | 0.8512 | 1.39 |
| rs3804857 | 7678617 | C | 0.1876 | 0.1947 | T | 0.1681 | 0.6818 | 0.9554 | 0.1112 | 0.7683 | 1.188 |
| rs1872397 | 7680410 | T | 0.1472 | 0.1396 | C | 0.2409 | 0.6236 | 1.064 | 0.1255 | 0.8316 | 1.36 |
| rs1872394 | 7680566 | A | 0.3318 | 0.334 | G | 0.01132 | 0.9153 | 0.9902 | 0.09253 | 0.826 | 1.187 |
| rs9819314 | 7681695 | T | 0.3309 | 0.332 | G | 0.003091 | 0.9557 | 0.9949 | 0.09263 | 0.8297 | 1.193 |
| rs11918634 | 7683434 | C | 0.2924 | 0.2853 | T | 0.1259 | 0.7227 | 1.035 | 0.09718 | 0.8556 | 1.252 |
| rs17673467 | 7685663 | C | 0.4426 | 0.4526 | T | 0.2103 | 0.6465 | 0.9605 | 0.08799 | 0.8083 | 1.141 |
| rs4686145 | 7686392 | A | 0.06342 | 0.07549 | C | 1.19 | 0.2753 | 0.8293 | 0.1718 | 0.5922 | 1.161 |
| rs4686146 | 7688564 | G | 0.3315 | 0.3471 | T | 0.5688 | 0.4507 | 0.9329 | 0.0921 | 0.7788 | 1.117 |
| rs4686148 | 7688662 | A | 0.3093 | 0.3248 | C | 0.5844 | 0.4446 | 0.9307 | 0.09393 | 0.7742 | 1.119 |
| rs3804850 | 7688702 | T | 0.2243 | 0.2264 | C | 0.01344 | 0.9077 | 0.988 | 0.1044 | 0.8051 | 1.212 |
| rs3864076 | 7708008 | G | 0.5046 | 0.4652 | A | 3.24 | 0.07185 | 1.171 | 0.08775 | 0.986 | 1.391 |
| rs11706732 | 7710584 | A | 0.431 | 0.4095 | T | 0.9762 | 0.3231 | 1.092 | 0.08952 | 0.9167 | 1.302 |
| rs2324209 | 7710841 | G | 0.06066 | 0.04804 | C | 1.626 | 0.2022 | 1.28 | 0.1938 | 0.8753 | 1.871 |
| rs9870241 | 7710922 | A | 0.05985 | 0.04536 | G | 2.199 | 0.1381 | 1.34 | 0.1978 | 0.9091 | 1.974 |
| SNP_A-2274307 | 7712944 | T | 0.06019 | 0.04519 | C | 2.353 | 0.1251 | 1.353 | 0.1978 | 0.9182 | 1.994 |
| rs3804843 | 7715397 | T | 0.05545 | 0.04331 | G | 1.641 | 0.2002 | 1.297 | 0.2035 | 0.8704 | 1.932 |
| rs9860395 | 7718570 | C | 0.06461 | 0.0507 | T | 1.839 | 0.1751 | 1.293 | 0.1901 | 0.891 | 1.877 |
| rs6782528 | 7721032 | A | 0.43 | 0.4479 | T | 0.6851 | 0.4078 | 0.9298 | 0.08792 | 0.7826 | 1.105 |
| rs162802 | 7721613 | G | 0.5018 | 0.4931 | A | 0.1594 | 0.6897 | 1.035 | 0.08729 | 0.8726 | 1.229 |
| rs162785 | 7727101 | T | 0.4788 | 0.4676 | C | 0.2641 | 0.6073 | 1.046 | 0.08742 | 0.8812 | 1.241 |
| rs329044 | 7748565 | G | 0.03676 | 0.02353 | T | 3.133 | 0.07674 | 1.584 | 0.262 | 0.9479 | 2.647 |
| rs9860560 | 7748736 | C | 0.4393 | 0.4422 | A | 0.01697 | 0.8964 | 0.9886 | 0.08778 | 0.8324 | 1.174 |
| rs1504047 | 7748843 | G | 0.4393 | 0.4422 | T | 0.01697 | 0.8964 | 0.9886 | 0.08778 | 0.8324 | 1.174 |
| rs332938 | 7753230 | A | 0.4384 | 0.4402 | G | 0.006749 | 0.9345 | 0.9928 | 0.08782 | 0.8358 | 1.179 |
| rs1155966 | 7757788 | G | 0.04926 | 0.05894 | A | 0.9606 | 0.327 | 0.8272 | 0.1938 | 0.5658 | 1.209 |
| rs1857697 | 7758107 | C | 0.4476 | 0.4549 | T | 0.113 | 0.7367 | 0.971 | 0.08758 | 0.8178 | 1.153 |
| rs1352411 | 7799831 | A | 0.07486 | 0.1023 | G | 4.697 | 0.03021 | 0.7102 | 0.1585 | 0.5206 | 0.9689 |
| rs1027527 | 7805160 | G | 0.1527 | 0.16 | A | 0.2093 | 0.6473 | 0.9462 | 0.121 | 0.7464 | 1.199 |
| rs6443124 | 7813180 | T | 0.1866 | 0.1853 | A | 0.005758 | 0.9395 | 1.009 | 0.112 | 0.8097 | 1.256 |
| rs6443125 | 7816412 | A | 0.1847 | 0.1853 | G | 0.001062 | 0.974 | 0.9963 | 0.1122 | 0.7996 | 1.242 |
| rs9841316 | 7820659 | T | 0.3408 | 0.3274 | G | 0.4196 | 0.5171 | 1.062 | 0.093 | 0.8851 | 1.274 |

BP, Position in base pairs of SNP based on NCBI build 37 (hg19); A1, Allele 1;F_A, Frequency of A1 in cases; F_U, Frequency of A1 in controls; A2, Allele 2; CHISQ, Basic allelic test chi-square (1df); P, Asymptotic p-value for this test; OR, Estimated odds ratio (for A1, i.e. A2 is reference); SE, Standard error; L95, Lower bound of 95% confidence interval for odds ratio; U95, Upper bound of 95% confidence interval for odds ratio.

**Supplementary table S2. Primer sequences used for screening *GRM7***

| **Primer name** | **Primer sequence** |
| --- | --- |
| GRM7_ExPa_F | CAAAGAAAGGCATTCCCAATCAATG |
| GRM7_ExPa_R | TGCTGACTGCTCCCAAACCC |
| GRM7_ExPb_F | GCGTCTTTAAGTCAAGAATCAGGCTC |
| GRM7_ExPb_R | TGCTGCTCGCTCTCTCCAACA |
| GRM7_Ex1a_F | CTGGGCTTTCCCGGAGGAG |
| GRM7_Ex1a_R | GGCTACCATGATGGAGACCGAA |
| GRM7_Ex1b_F | GGAAGCGATGCTCTACGCCC |
| GRM7_Ex1b_R | TGTCAAGCCGGATGGAGTGG |
| GRM7_Ex2_F | TCCCATGCTAACCTCCTTCCC |
| GRM7_Ex2_R | CAGGTGTACTTCACAGGACTCTGGG |
| GRM7_Ex3_F | TCCTCGCTTAGCAACAGCATCA |
| GRM7_Ex3_R | CAAGCACAGACTTCGCCGCT |
| GRM7_Ex4_F | TTGCTCTTGTTTCTGAAGATGC |
| GRM7_Ex4_R | TTTGAAACAACCCCACATGA |
| GRM7_Ex5_F | TTTCGGTCGACACACAAGAGGA |
| GRM7_Ex5_R | CCTGGCTGGAACTGAACAAGAGG |
| GRM7_Ex6_F | TCGAAGCAGTGTTTTCTTTAAGC |
| GRM7_Ex6_R | CCAAACCATAACCCCCAAAT |
| GRM7_Ex7_F | GGGGACCTATTAAAGGGGATA |
| GRM7_Ex7_R | CCACAACAAATTGCATGCTC |
| GRM7_Ex8a_F | TCTTGCAAGCTGAGGGTAAA |
| GRM7_Ex8a_R | GATGCCCGTCAAAAGAACAT |
| GRM7_Ex8c_F | GGGATCATTGCCACCATCTTTG |
| GRM7_Ex8c_R | ATCCAATGGGCTTGGCTTCG |
| GRM7_Ex8d_F | GCCCAACATCACAACTGGCAA |
| GRM7_Ex8d_R | TCCAGTGGAATGAACGTGGCTC |
| GRM7_Ex9_F | GCATGTGCATGGATTGACCC |
| GRM7_Ex9_R | TGGCATTCACCAAGGCAACC |
| GRM7_Ex10_F | TGTTTGGGCTGGTTCCCTCA |
| GRM7_Ex10_R | GGCAGCTGCTGGCACTAAGC |
| GRM7_Ex11_12_F | CCAAGGTCAGTTAATCTCCAAAA |
| GRM7_Ex11_12_R | GGGACTCCCAGTCCAGTTT |
| GRM7_Ex13_F | GAAACTGGCCACCAGGGTCA |
| GRM7_Ex13_R | GGATGTTGCTGGCATGTGATGA |
| GRM7_Ex14_F | TGCAGGAAGAGTAGCATTCATGGG |
| GRM7_Ex14_R | GGCTTTAAGGCATCATTGCTCCA |
| grm7_15a_F1 | CTTAGAGTCAGAGGAATC |
| grm7_15a_R1 | GCTCTCAGTGGTCCATTC |
| GRM7_Ex15c_F | TCACTGACATCAGCACTGCCAA |
| GRM7_Ex15c_R | GGCATGCATTCCAAAGATCAAGA |
| GRM7_Ex15d_F | CCACTGCACATCATGTTTTT |
| GRM7_Ex15d_R | TCTTGGACAATATCACAGAATTGAA |
| GRM7_Ex15e_F | CGTTAATCTTGCTGCTTATGTGCCA |
| GRM7_Ex15e_R | CCTCTTCCCTTCCCACTTGCTG |
| GRM7_Ex3a_F | ATGGTCCTGTCTTTGCGTTC |
| GRM7_Ex3a_R | TTTAGTTTTATTCCACTTATTCCACTG |
| GRM7_Ex3b_F | CTTGGACGGCCATTAGGACT |
| GRM7_Ex3b_R | CCACAATAAAGAAACTTGAACAGG |
| GRM7_Ex3c_F | AAAAATGTTATTTGTATGGCTCAAAA |
| GRM7_Ex3c_R | CATCTTACTTCCCTGCTTCTTG |
| GRM7_Ex3d_F | TTTTCACAATCATGCTCTGCT |
| GRM7_Ex3d_R | TTCTTTAAAATATAAACACCCAACAA |
| GRM7_Ex3e_F | ACGACCAATTTGGCATTTTTA |
| GRM7_Ex3e_R | AAGAAACATCTAAAATCAGCAACG |
| GRM7_Ex3f_F | TTCAAATAATAAATTAGCCTTTTGTTC |
| GRM7_Ex3f_R | AAGATACAAAGTACCAAAACTGAATGC |
| GRM7_Ex3g_F | GCAACCTTGTTTAAGGCACT |
| GRM7_Ex3g_R | CAGGAAAAGATATTACCAAACAAAA |
| grm7_9a_F1 | GCATCAGGAAGCTCTAGAAG |
| grm7_9a_R1 | GATGCCGTTATCATGGTAGTG |
| GRM7_Ex9b_F | TTCCTCTAGCATGTGAATGTTGA |
| GRM7_Ex9b_R | AAATCCCTTCTGCCTTAAACAA |
| GRM7_Ex9c_F | TTGCATTTAGAATGAGGCAGA |
| GRM7_Ex9c_R | ACACTGAATGCCACAGCAAG |
| GRM7_Ex9d_F | ATTGTGGGCTCCAGTTTGAC |
| GRM7_Ex9d_R | ACGCCCAGCCATAAATTCTT |
| GRM7_Ex9e_F | TTCCTGAAGAGGCAAAAAGC |
| GRM7_Ex9e_R | TGCAAATTTGACAAGACAACC |
| GRM7_Ex9f_F | GGCAACAAGAGCAAAACTCC |
| GRM7_Ex9f_R | CACACAAAGCATTCCGCATA |
| GRM7_Pd_F | CCAGATGGGGAAACTAAAACC |
| GRM7_Pd_R | GAAAGAGGTGATGGCAGGAA |
| GRM7_Pe_F | TTATCCCTGAATGGCTCTGG |
| GRM7_Pe_R | TGGCAATGTAACGAAGAAACC |
| GRM7_Pf_F | TCATTCTTTATCTTTCTCTATCTCCA |
| GRM7_Pf_R | TGCCCCTCTTACTTGTTTCAA |
| GRM7_Pg_F | CGAGACATGTGCAGGTGAAA |
| GRM7_Pg_R | GTACCTGCTCCTTCCTGCAA |
| GRM7_Ph_F | TCCAGAAACTGTCTATAGCGACTAA |
| GRM7_Ph_R | AGCACCTCAGGCTGTCTTTG |
| grm7_nPa_F2 | GACAGTGCTATAAGCAAGATTTG |
| GRM7_isonew_nPa_R1 | CCAGATCCCTTTCACTGGTC |
| grm7_nPb_F1 | AGCACAGTGGCTGTCCGG |
| grm7_nPb_R1 | GCTCAGTCGCAATTCAAC |
| GRM7_isonew_nex4_F | GGTTGGCAAGAAAGATTGGA |
| GRM7_isonew_nex4_R | AATGCTCTTTTGGCCAGGAT |
| GRM7_isonew_nex5_F | AAGGATGCTTAAGGACCAACTG |
| GRM7_isonew_nex5_R | CCACAAAGCTAAGAGAGAAGTGG |
| GRM7_isonew_nex7_F | AGTTACCTTTCCTGCTCCTCTC |
| GRM7_isonew_nex7_R | AGTGGAAAGCGATTCCTCAA |
| GRM7_isonew_nex8_F | GCCTAAACTGTGTCCACTAGGC |
| GRM7_isonew_nex8_R | CCACTTGCTTTATGGGGAAG |

**Supplementary table S3**. Primers for genotyping GRM7 SNPs using Kaspar and HRM assay

| **Primer ID** | **Primer sequence** |
| --- | --- |
| rs114774914_F1 | GAAGGTGACCAAGTTCATGCTCAGGATGCCAGTACAGGTTGCA |
| rs114774914_F2 | GAAGGTCGGAGTCAACGGATTCAGGATGCCAGTACAGGTTGCT |
| rs114774914_R1 | CAGGAGGAGACGCCGCTCATAA |
| rs56173829_F1 | GAAGGTGACCAAGTTCATGCTAGGTACGACATCAGATGGCAAAGT |
| rs56173829_F2 | GAAGGTCGGAGTCAACGGATTAGGTACGACATCAGATGGCAAAGA |
| rs56173829_R2 | CTAGGTTGCAAGGTTTTGAAATTTTCTGTA |
| rs17726576_F1 | GAAGGTGACCAAGTTCATGCTGTTTGTTTTCGAATGCCTTGTTTTC |
| rs17726576_F2 | GAAGGTCGGAGTCAACGGATTCACTGTTTGTTTTCGAATGCCTTGTTTTT |
| rs17726576_R1 | ATTCCACCGTCTGAGAGAATA |
| rs342034_F1 | GAAGGTGACCAAGTTCATGCTCCCCGGAGGGAGCGCAC |
| rs342034_F2 | GAAGGTCGGAGTCAACGGATTGCCCCGGAGGGAGCGCAT |
| rs342034_R2 | AACATCCTGAGGCTCTTCCAGGTA |
| 3_6901914_F1 | GAAGGTGACCAAGTTCATGCTCCATCACCTCTTTCCTGCTCTTT |
| 3_6901914_F2 | GAAGGTCGGAGTCAACGGATTCCATCACCTCTTTCCTGCTCTTA |
| 3_6901914_R2 | CTGGTGCACCTGGTGGCAGTTT |
| GRM7_Ex3f_7313045_F1 | GAAGGTGACCAAGTTCATGCTAATACCTAAACCAGGAAAAGATATTACCA |
| GRM7_Ex3f_7313045_F2 | GAAGGTCGGAGTCAACGGATTACCTAAACCAGGAAAAGATATTACCG |
| GRM7_Ex3f_7313045_R2 | GCAAATATGTTCCAAGTAATATTGTCTATA |
| GRM7_rs35106713_Ex2_F1 | GAAGGTGACCAAGTTCATGCTGAAGTCATAGCGCCGGTCATCA |
| GRM7_rs35106713_Ex2_F2 | GAAGGTCGGAGTCAACGGATTAAGTCATAGCGCCGGTCATCG |
| GRM7_rs35106713_Ex2_R2 | GCATCAACGGCACCCGAGCTAA |
| rs140139253_F1 | GAAGGTGACCAAGTTCATGCTGAGAACTCTACAAGCCACAGACG |
| rs140139253_F2 | GAAGGTCGGAGTCAACGGATTAAGAGAACTCTACAAGCCACAGACA |
| rs140139253_R2 | CTCTGATCAAGCTGGCTCTCTTCAA |
| rs138571076_F1 | GAAGGTGACCAAGTTCATGCTGTTGTTTAATTTCCAAGTGTTTGGAGAT |
| rs138571076_F2 | GAAGGTCGGAGTCAACGGATTGTTGTTTAATTTCCAAGTGTTTGGAGAC |
| rs138571076_R4 | CAAAGTAGAAATCAATGACAGAAAGATAAA |
| rs192193072_F1 | GAAGGTGACCAAGTTCATGCTATATTTGTAGAAAAGTCCTAATGGCCG |
| rs192193072_F2 | GAAGGTCGGAGTCAACGGATTAAATATTTGTAGAAAAGTCCTAATGGCCA |
| rs192193072_R2 | CCCCTGGGTCTTGTGCCCCTT |
| rs721774_F1 | GAAGGTGACCAAGTTCATGCTGAATGTGCCAGCAGTGCCCC |
| rs721774_F2 | GAAGGTCGGAGTCAACGGATTGAATGTGCCAGCAGTGCCCT |
| rs721774_R1 | CCCTAAACAGAGTTAAATGAAAAGGTCATT |
| nPb_7467774_F1 | GAAGGTGACCAAGTTCATGCTCTTATTGTTTCTTCACTACACAAATG |
| nPb_7467774_F2 | GAAGGTCGGAGTCAACGGATTCTCTTATTGTTTCTTCACTACACAAATA |
| nPb_7467774_R2 | CCAAATTGTTTCCTACGGCA |
| rs1965222_F1 | GAAGGTGACCAAGTTCATGCTGATGTTAGAAGGATGGGATAGAGACT |
| rs1965222_F2 | GAAGGTCGGAGTCAACGGATTATGTTAGAAGGATGGGATAGAGACC |
| rs1965222_R1 | CTGGAAGGACTTTCCATGCCAACAT |
| GRM7_9c_7698252_F1 | GAAGGTGACCAAGTTCATGCTACTTGTCCTAGGACTTCTGTCAC |
| GRM7_9c_7698252_F2 | GAAGGTCGGAGTCAACGGATTAGACTTGTCCTAGGACTTCTGTCAT |
| GRM7_9c_7698252_R2 | CAACATTGTGGGCTCCAGTTTGACTT |
| GRM7_15e_7758348_F1 | GAAGGTGACCAAGTTCATGCTGGTTTTTAATATGCCTTTCAGAAATGGC |
| GRM7_15e_7758348_F2 | GAAGGTCGGAGTCAACGGATTGGGTTTTTAATATGCCTTTCAGAAATGGT |
| GRM7_15e_7758348_R2 | CAAGTAACATGCTTTAGCTCACGACTA |
| Pa_6877167_GRM7 | Pre-designed Kaspar assay from Kbiosciences, UK |
| rs2229902 | Pre-designed Kaspar assay from Kbiosciences, UK |
| rs140995942_F_HRM | GTTAGGAGTTGGTGACCAG |
| rs140995942_R_HRM | TGGAGTTTTGCTCTTGTTGC |

**Supplementary table S4**. Primers for validating CNVs in GRM7 using RNase P CNR assay

| **Primer ID** | **Sequence** | **Roche UPL probe** |
| --- | --- | --- |
| GRM7_CNV_p19_F1 | GCATAGCTGACAGAAGGAAGC | 19 |
| GRM7_CNV_p19_R1 | TGTCCTGCAAGACGTATGCT |
| GRM7_CNV_p19_F2 | AGACAGAGTCCCACTCTCTTGG | 19 |
| GRM7_CNV_p19_R2 | TGTGGTGAGCCAAAATTGC |
| GRM7_CNV_p22_F1 | AGTCGGCAGCCTTGTTAAAG | 22 |
| GRM7_CNV_p22_R1 | TGTACCACCTTGGTTTCACACT |
| GRM7_CNV_p38_F1 | CGCTTATTTATCACCCGTTCC | 38 |
| GRM7_CNV_p38_R1 | TTGAAGAGGCATCTACCTGGA |

**Supplementary table S5. SNPs found by resequencing *GRM7***

| **Region** | **SNP ID** | **Position (NCBI35/hg19)** | **Alleles** | **MAF** |
| --- | --- | --- | --- | --- |
| Promoter | 3_6900524_1000G | 6900524 | CT/C | 0.18* |
| Promoter | rs183111337 | 6900527 | T/C | 0.01* |
| Promoter | rs62237226 | 6900528 | C/T | 0.19* |
| Promoter | rs115717493 | 6900573 | G/A | 0.01* |
| Promoter | rs340653 | 6900663 | C/T | 0.3* |
| Promoter | rs163422 | 6900744 | C/T | 0.08* |
| Promoter | rs371097 | 6900910 | C/A | 0.1* |
| Promoter | rs371841 | 6900912 | C/T | 0.29* |
| Promoter | rs62237227 | 6901071 | C/T | 0.19* |
| Promoter | rs339807 | 6901090 | G/A | 0.26 |
| Promoter | rs63470962 | 6901176 | C/CAAG | 0.09* |
| Promoter | rs114774914 | 6901783 | A/T | 0.03* |
| Promoter | 3_6901914_1000G | 6901976 | AAGA/- | NA |
| Promoter | rs62237228 | 6902167 | A/G | 0.19* |
| Promoter | 3_6902624_1000G | 6902624 | T/TC | 0.25* |
| Exon 1 | rs3749380 | 6903297 | C/T | 0.45 |
| Exon 1 | rs342034 | 6903601 | A/G | 0.04 |
| Exon 2 | rs3828472 | 7188006 | G/T | 0.33 |
| Exon 2 | rs9868134 | 7188033 | T/C | 0.22 |
| Exon 2 | rs3749450 | 7188063 | G/A | 0.33 |
| Exon 2 | rs3749449 | 7188072 | A/G | 0.18 |
| Exon 2 | rs3749448 | 7188116 | G/A | 0.18 |
| Exon 2 | rs35106713 | 7188180 | T/C | 0.02 |
| Exon 2 | rs140139253 | 7188396 | T/C | 0.001* |
| Promoter of ENST00000463676 isoform | rs6768211 | 7337515 | G/T | NA |
| Promoter of ENST00000463676 isoform | rs185220306 | 7337786 | T/G | 0.01* |
| Promoter of ENST00000463676 isoform | rs9850091 | 7337788 | G/T | 0.44* |
| Promoter of ENST00000463676 isoform | rs116361217 | 7337904 | C/T | 0.07* |
| Promoter of ENST00000463676 isoform | GRM7_3f_7313045 | 7338045 | G/A | NA |
| Promoter of ENST00000463676 isoform | rs7638548 | 7338112 | G/A | 0.37* |
| Promoter of ENST00000463676 isoform | rs78210196 | 7338179 | C/A | 0.01* |
| Promoter of ENST00000463676 isoform | rs7640834 | 7338353 | G/A | 0.37* |
| Promoter of ENST00000463676 isoform | rs61060115 | 7338521 | -/A | NA * |
| Promoter of ENST00000463676 isoform | rs75618003 | 7338776 | C/T | 0.18* |
| Promoter of ENST00000463676 isoform | rs138571076 | 7338985 | A/G | 0.01* |
| Promoter of ENST00000463676 isoform | rs138761134 | 7339328 | ATGTT/A | 0.01* |
| Promoter of ENST00000463676 isoform | rs78677232 | 7339409 | C/T | 0.18* |
| Promoter of ENST00000463676 isoform | rs192193072 | 7339471 | A/G | 0.002* |
| Promoter of ENST00000463676 isoform | rs2291867 | 7340164 | C/T | 0.39 |
| Exon 5 | rs712774 | 7456675 | C/T | 0.26 |
| Promoter of ENST00000458641 isoform | GRM7_nPb_7467774 | 7492774 | C/T | NA |
| Promoter of ENST00000458641 isoform | rs712792 | 7492779 | C/T | 0.26 |
| Promoter of ENST00000458641 isoform | nPa_7493030 | 7493030 | -/C indel | NA |
| Promoter of ENST00000458641 isoform | rs73015547 | 7493347 | C/T | 0.13* |
| Promoter of ENST00000458641 isoform | rs712793 | 7493351 | A/G | 0.34 |
| Exon 6 | rs2229902 | 7494417 | A/T | 0.44 |
| Exon 8 | rs1485174 | 7595828 | A/G | 0.29 |
| Exon of ENST00000458641 isoform | nex4_7600830 | 7600830 | T indel | NA |
| Exon of ENST00000458641 isoform | rs1143739 | 7601039 | G/T | 0.26 |
| Exon of ENST00000458641 isoform | rs1965222 | 7603194 | C/T | 0.13 |
| Exon of ENST00000458641 isoform | rs3828429 | 7603367 | C/G | 0.01* |
| Exon 8 | rs34373930 | 7620168 | A/G | 0.3* |
| Exon 8 | rs7614915 | 7620382 | C/T | 0.26 |
| Exon 8 | rs1485175 | 7620789 | C/T | 0.43 |
| Exon 8 | rs1485173 | 7621093 | A/T | 0.3* |
| Exon 8 | rs1485172 | 7621158 | C/T | 0.43 |
| Exon of ENST00000458641 isoform | rs2139187 | 7649591 | G/T | 0.34* |
| Exon of ENST00000458641 isoform | nex7_7649745 | 7649745 | Insertion of T | NA |
| Exon of ENST00000458641 isoform | rs114582026 | 7649815 | G/T | 0.16* |
| Exon of ENST00000458641 isoform | rs115155482 | 7649819 | C/T | 0.3* |
| Exon of ENST00000458641 isoform | rs7611935 | 7678010 | A/G | 0.34* |
| Exon of ENST00000458641 isoform | rs7612048 | 7678090 | A/G | 0.3 |
| Exon of ENST00000458641 isoform | rs17047754 | 7678134 | C/T | 0.3 |
| Exon of ENST00000458641 isoform | nex8_7678357 | 7678357 | G/T | NA |
| Exon 9 | rs162802 | 7721613 | T/C | 0.5 |
| Exon 9 | rs2280739 | 7721997 | G/A | 0.06 |
| Exon 9 3' UTR | rs10514663 | 7723179 | G/A | 0.08 |
| Exon 9 3' UTR | GRM7_9c_7698252 | 7723252 | G/A | NA |
| Exon 9 3' UTR | rs162801 | 7723744 | G/A | 0.5* |
| Exon 9 3' UTR | rs140995942 | 7723896 | G/A | 0.01* |
| Exon 9 3' UTR | STR | 7724024 | Simple T repeat |  |
| Exons 11 and 12 | rs5846531 | 7732725 | -/T | 0.02* |
| Exon 14 | rs2279840 | 7735770 | A/G | 0.2 |
| Exon 14 | Ex14_Indel | 7736114 | 18 bp insertion | NA |
| Exon 14 | rs162777 | 7736246 | C/T | 0.26 |
| Exon 15, 3' UTR | rs75721571 | 7782367 | C/G | 0.07* |
| Exon 15, 3' UTR | rs9826579 | 7782371 | C/T | 0.24* |
| Exon 15, 3' UTR | rs56173829 | 7782494 | A/T | 0.01* |
| Exon 15, 3' UTR | rs17726576 | 7782551 | C/T | 0.02 |
| Exon 15, 3' UTR | rs60445645 | 7782703 | -/T | NA |
| Outside the gene, 3' end | rs150288969 | 7783347 | G/A | 0.01* |

MAF, minor allele frequency; NA, not available; *MAF derived from the 1000 genomes project

**Supplementary table S6. Significantly associated (*P* ≤ 0.05) *GRM7* SNPs using imputation from 1000G *GRM7*** SNPs

| **SNP** | **Position** | **A1** | **A2** | **GENO** | **NPRX** | **INFO** | **F_A** | **F_U** | **OR** | **P** |
| --- | --- | --- | --- | --- | --- | --- | --- | --- | --- | --- |
| rs10510354 | 7252190 | C | G | 0.408 | 2 | 0.479 | 0.375 | 0.408 | 0.87 | 0.003 |
| rs1352411 | 7799831 | A | G | 0.389 | 2 | 0.423 | 0.079 | 0.092 | 0.839 | 0.017 |
| rs340659 | 6906622 | C | T | 0.408 | 2 | 0.54 | 0.065 | 0.052 | 1.27 | 0.021 |
| rs11708019 | 7229619 | G | A | 0.408 | 2 | 0.441 | 0.343 | 0.365 | 0.908 | 0.027 |
| rs9823996 | 7244509 | G | C | 0.408 | 2 | 0.475 | 0.316 | 0.294 | 1.11 | 0.028 |
| rs9820417 | 7104786 | G | A | 0.387 | 2 | 0.438 | 0.238 | 0.258 | 0.899 | 0.033 |
| rs17288442 | 6866379 | A | C | 0.407 | 2 | 0.479 | 0.302 | 0.324 | 0.903 | 0.034 |
| rs13070476 | 7259313 | C | T | 0.403 | 2 | 0.45 | 0.226 | 0.245 | 0.901 | 0.034 |
| rs1508724 | 7241745 | A | G | 0.763 | 2 | 0.82 | 0.311 | 0.284 | 1.14 | 0.035 |
| rs6769814 | 7251433 | G | A | 0.763 | 2 | 0.802 | 0.324 | 0.297 | 1.13 | 0.035 |
| rs17047149 | 7380288 | G | A | 0.407 | 2 | 0.353 | 0.007 | 0.011 | 0.623 | 0.052 |
| rs11717750 | 7464013 | T | C | 0.408 | 2 | 0.846 | 0.507 | 0.479 | 1.12 | 0.053 |

A1, Allele 1; A2, Allele 2; GENO, Genotyping for the reference SNP; NPRX, Number of proxy SNPs used to tag reference SNP; INFO, Information metric for each reference SNP; F_A; Reference SNP allele frequency in cases; F_U; Reference SNP allele frequency in controls; OR, odds-ratio; P, Asymptotic p-value for test of association. All the positions are correct according to NCBI35/hg19 assembly

**Supplementary table S7. CNVs in GRM7 identified in UCL1 GWAS (McQuillin et al., 2011)**

| **Location** | **CNV** | **Sample** |
| --- | --- | --- |
| Chr3:7347535 - 7733108 | Duplication | Case |
| Chr3:7277350 - 7558603 | Deletion | Case |
| Chr3:7001273 - 7107443 | Deletion | Case |
| Chr3:7040496 - 7208936 | Deletion | Control |
